# Transcranial direct current stimulation of the medial prefrontal cortex dampens mind-wandering in men

**DOI:** 10.1038/s41598-017-17267-4

**Published:** 2017-12-05

**Authors:** Elena Bertossi, Ludovica Peccenini, Andrea Solmi, Alessio Avenanti, Elisa Ciaramelli

**Affiliations:** 1 Dipartimento di Psicologia e Centro studi e ricerche in Neuroscienze Cognitive, Università di Bologna, Bologna, Italy; 2 IRCCS Fondazione Santa Lucia, Roma, Italy

## Abstract

Mind-wandering, the mind’s capacity to stray from external events and generate task-unrelated thought, has been associated with activity in the brain default network. To date, little is understood about the contribution of individual nodes of this network to mind-wandering. Here, we investigated the role of medial prefrontal cortex (mPFC) in mind-wandering, by perturbing this region with transcranial direct current stimulation (tDCS). Young healthy participants performed a choice reaction time task both before and after receiving cathodal tDCS over mPFC, and had their thoughts periodically sampled. We found that tDCS over mPFC - but not occipital or sham tDCS - decreased the propensity to mind-wander. The tDCS-induced reduction in mind-wandering occurred in men, but not in women, and was accompanied by a change in the content of task-unrelated though, which became more related to other people (as opposed to the self) following tDCS. These findings indicate that mPFC is crucial for mind-wandering, possibly by helping construction of self-relevant scenarios capable to divert attention inward, away from perceptual reality. Gender-related differences in tDCS-induced changes suggest that mPFC controls mind-wandering differently in men and women, which may depend on differences in the structural and functional organization of distributed brain networks governing mind-wandering, including mPFC.

## Introduction

Mind-wandering occurs when attention shifts away from an ongoing task or events in the external environment towards self-generated thoughts unrelated to perceptual reality, and centered instead on current concerns, memories, and future experiences^1^. Common examples are replaying mentally your last meeting with a friend while attending a class, or simulating an approaching job interview while swimming. Humans generally spend a considerable amount of time mind-wandering^2,3^, though gender effects have been reported, with women reporting more vivid mental imagery in mind-wandering than men^4^ (see also^5^). Women, compared to men, also show a higher susceptibility for ruminative thinking, the persistent replay of (negative) thoughts unrelated to the “here and now”, which is related to - if not an instance of - mind-wandering^6^.

It has been shown that mind-wandering brings some benefits: it may favor creativity and self-reflection, and reduce impulsivity during (intertemporal) choice^1,7^. Mind-wandering, however, is not costless: it impairs processing of external events^8^, and disturbs performance in ongoing activities^1^. Moreover, clinical psychologists have long considered daydreaming a hallmark for mental illness, and a manifestation of frustration^9^. Mind-wandering is not only a consequence, but also a cause of low mood. Killingsworth and Gilbert have found that mind-wandering was negatively related to happiness in the moment, and a time-lag analysis led them to conclude that mind-wandering results in bad mood^3^. Mar and colleagues also found a negative relation between daydreaming and happiness, though this association was driven by the frequency of mind-wandering in men, and by its vividness in women^5^.

Given the pervasiveness of mind-wandering and its important impact on humans’ mental life, much research is being dedicated to reveal the cognitive and neural mechanisms governing mind-wandering. Functional magnetic resonance imaging (fMRI) evidence indicates that mind-wandering is associated with activity in the ‘default network’, a set of interconnected brain regions including the medial prefrontal cortex (mPFC), the posterior cingulate cortex, the angular gyrus, and the medial temporal lobes (MTLs) bilaterally, whose activity is enhanced during internally focused thought^10–12^. Activity in the default network has been linked to the production of mental contents that generally populate mind-wandering episodes, such as remembered or simulated experiences involving the self and others^1,13–15^. Indeed, remembering the past, imagining the future, and conceiving the perspective of other people all activate the default network^16^. Mind-wandering is also associated with activity in the ‘executive network’, including the dorsolateral and ventrolateral prefrontal cortex^10^, which is positively correlated with that of the default network, suggesting a functional interplay of the two networks for mind-wandering^1,17^.

Although there is abundant fMRI evidence for the involvement of the default network and the executive network in mind-wandering, imaging techniques provide correlational evidence, and cannot establish a direct, causal link between brain and function. To test the causal role of specific nodes of the neural networks underlying mind-wandering, it is therefore fundamental to recur to causal methods, for example investigating the effect of transient non-invasive neurostimulation or stable brain lesions on the propensity to mind-wander^18^. Recently, two independent research groups have used transcranial direct current stimulation (tDCS) to target a key region of the executive network, the dorsolateral prefrontal cortex (dlPFC), and modulate mind-wandering. In a first study, Axelrod and colleagues administered tDCS (at 1 mA and for 20 min) with the anodal electrode over the left dlPFC (i.e., over the F3 electrode of the 10–20 EEG system) and the cathodal electrode over the right supraorbital area^19^. Anodal tDCS over dlPFC (supposedly upregulating dlPFC activity) increased the propensity to mind-wander relative occipital or sham stimulation^19^. Kajimura and Nomura administered tDCS (at 1.5 mA for 20 min) with the cathodal electrode over left dlPFC (i.e., over the AF7 electrode) and the anodal electrode over right parietal regions (i.e., over the P4 electrode)^20^. This stimulation decreased the propensity to mind-wander relative to the reverse montage (cathodal-parietal/anodal-frontal)^20^. Together, these studies indicate that left dlPFC plays a crucial role in mind-wandering. In these studies, however, the use of a cephalic reference over the right hemisphere may have caused a possible contribution of interhemispheric imbalance to the observed behavioral effects.

In the present research, we used tDCS to test the functional relevance of the mPFC, a main component of the default network^13^, to mind-wandering. There are several reasons to hypothesize that mPFC plays an essential role in mind-wandering. mPFC is consistently engaged in association with mind-wandering^10,11,21,22^. Moreover, in healthy individuals, the thickness of mPFC is positively related to the tendency to mind-wander under low-demanding conditions^7^. Importantly, Bertossi and Ciaramelli found reduced mind-wandering in patients with lesions to the ventral portion of mPFC compared to healthy controls and control patients with posterior lesions^23^. They also found that ventral mPFC patients’ (infrequent) mind-wandering episodes focused less on the future and more on the present than those of the control groups^23^. These results suggest that ventral mPFC plays a crucial role in mind-wandering, possibly by mediating the construction of future-related thoughts sufficiently vivid to divert attention inward, away from the task at hand. Yet, this study left unanswered the fundamental question of whether exogenous manipulation of intact mPFC in healthy participants would affect their propensity to mind-wander and the content of task-unrelated thought. Moreover, although behavioral research has documented gender differences in mind-wandering^4^ (see also^5,6^) whether men and women show a different sensitivity to tDCS effects on mind-wandering is an unexplored question.

**Figure 1:**
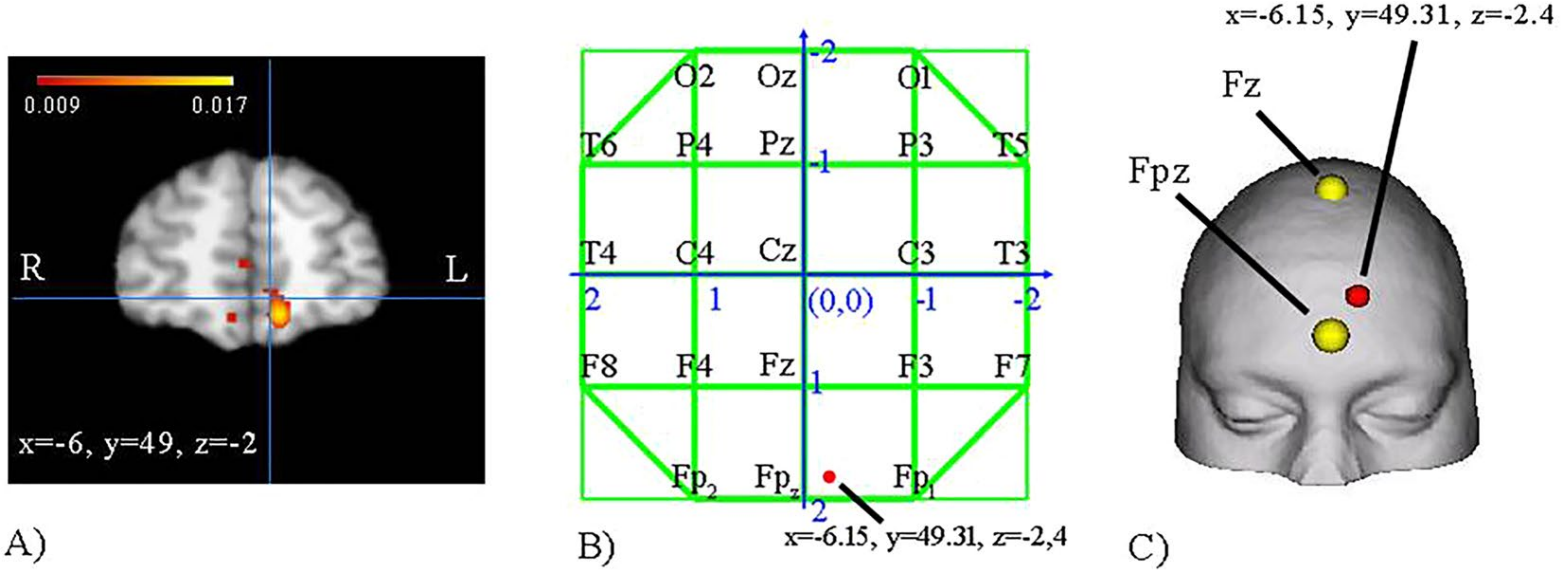
Panel (A) Coronal view of ALE map, showing the peak of the meta-analytic cluster of activation for mind-wandering in BA 10; color bar represents ALE values; R: right; L: left. Panel (B) The peak of the meta-analytic cluster of activation for mind-wandering in BA 10 on the 2D map of the 10–20 system. Panel (C) 3D representation of the position of the meta-analytic cluster of activation for mind-wandering in BA 10.

Here, we investigate the role of mPFC in mind-wandering by targeting this region in healthy young female and male participants using cathodal tDCS, which is thought to decrease cortical excitability^24^. While our previous study on brain-lesioned patients involved patients with focal lesions to the ventral mPFC^23^ – and therefore necessarily speaks to the critical role of the ventral portion of mPFC in mind-wandering – here we conducted a preliminary activation likelihood estimation (ALE) meta-analysis (see^25,26^) of neuroimaging studies of mind-wandering to select the mPFC sector most consistently associated with mind-wandering in healthy participants, and made it the target region for tDCS (see Fig. 1 and Methods). We administered cathodal currents for 15 minutes at 2 mA (25 cm² electrode; current density ~0.08 mA/cm^2^) over the mPFC, to alter its neural functioning for several minutes after the end of the stimulation^27,28^. Importantly, to avoid possible confounding effects deriving from a cephalic reference electrode, we used a monopolar montage with the anodal electrode applied over the deltoid muscle^27–29^. We tested three groups of 24 participants, matched for demographical variables and well as working memory capacity and tendency towards daydreaming in daily life (see Table 1 and Methods), who performed a simple choice reaction time task (CRT)^14,23^ before and after the tDCS stimulation. The CRT task consisted in monitoring a stream of digits written in black ink or, rarely, in green ink. Upon presentation of a green digit, participants had to report whether that number was even or odd^14,23^ (see Methods). Participants’ thoughts during the CRT task were periodically sampled with thought probes requiring to rate the degree to which, immediately before the probe, their attention was on-task vs. off-task, and to classify the content of off-task thoughts (see Methods). One group received cathodal tDCS over mPFC (mPFC group), another group received cathodal tDCS over a control site in the occipital cortex, which is not part of the default network^15^ (occipital group), and the last group received sham tDCS (sham group). If mPFC is crucial for mind-wandering, then cathodal tDCS over mPFC should reduce mind-wandering compared to sham tDCS or tDCS over the occipital cortex. To test for gender differences in tDCS-induced changes in behavior, each tDCS group comprised 12 women and 12 men.

**Table 1:** Demographic information and cognitive performance.

		N	Age	Education	Daydreaming scale	3-back (accuracy)	3-back (RTs)
Female	mPFC	12	23.42(0.54)	17.08(0.36)	45.58(1.79)	0.67(0.08)	817.30(52.57)
	occipital	12	22.17(0.34)	16.92(0.26)	48.00(1.60)	0.74(0.05)	804.34(38.77)
	sham	12	23.42(0.67)	16.67(0.56)	45.50(2.05)	0.68(0.05)	852.14(76.26)
Male	mPFC	12	23.50(0.42)	16.92(0.38)	42.25(1.76)	0.74(0.06)	882.56(62.43)
	occipital	12	22.58(0.65)	16.42(0.51)	44.00(3.75)	0.76(0.06)	865.53(81.63)
	sham	12	23.75(0.66)	17.00(0.33)	42.00(2.61)	0.74(0.06)	835.22(49.51)

Note. mPFC = participant group receiving cathodal tDCS on the medial prefrontal cortex; occipital = participant group receiving cathodal tDCS on the occipital cortex; sham = participant group receiving sham stimulation; RTs = reaction times. Values in parentheses are standard errors of the mean.

## Results

### Daydreaming

Participant groups’ general tendency towards daydreaming was assessed with the daydreaming scale of the Imaginal Processes Inventory (IPI)^30^ (see Methods). Table 1 shows daydreaming frequency scores attained at the IPI^30^. Daydreaming scores were non-normally distributed (Kolmogorov-Smirnov test, d = 0.17, p < 0.05), and, therefore, were analyzed with non-parametric tests. A Kruskal-Wallis ANOVA with Stimulation as factor (mPFC, occipital, sham) showed no significant effect (H = 3.17, p = 0.21), assuring that stimulation groups were matched in terms of general proclivity towards mind-wandering. We obtained a similar null result when we used a Mann-Whitney Z test with Gender as factor (males, females) across stimulation groups (Z = 1.47, p = 0.14). Lastly, no significant differences in self-reported daydreaming were found when considering the six groups in the Kruskal-Wallis ANOVA (mPFC-men, mPFC-women, occipital-men, occipital-women, sham-men, sham-women; H = 5.46, p = 0.36).

### Working memory

Participant groups’ working memory abilities were assessed with a 3-back task^31^ (see Methods). Accuracy in the 3-back task (hit rates – false alarms rates), and reaction times (RTs) for correct responses (see Table 1) were analyzed using a Stimulation (mPFC, occipital, sham) x Gender (men, women) ANOVA. We found no significant effect of stimulation, gender, or their interaction in working memory accuracy (all F < 0.85, all p values > 0.36) or RTs (all F < 0.52, all p > 0.47). These findings ensure that the different participant groups were well matched for working memory abilities.

### Effect of tDCS on mind-wandering

#### Accuracy and reaction times in the CRT task

Mean accuracy and RTs for correct responses in the CRT task were calculated separately for each participant before and after tDCS. Table 2 shows mean accuracy and RTs by participant group and session. We first ran a Stimulation (mPFC, occipital, sham) x Gender (male, female) x Session (before tDCS, after tDCS) ANOVA on accuracy. We found no significant main effects or interactions (F < 3.24, p > 0.077 in all cases). The same ANOVA on RTs indicated a main effect on Session (F_(1,66)_ = 10.34, p = 0.002, partial η^2^ = 0.14), such that all participant groups became slower at responding in the post-tDCS compared to the pre-tDCS session (883.06 vs. 847.69, Cohen’s d = 0.23), presumably as a result of tiredness. No other effects on RTs were significant (F < 1.48, p > 0.23 in all cases). These findings indicate that participant groups had similar baseline levels of performance in the ongoing (CRT) task before tDCS, and that tDCS had no effect on performance in this task.

**Table 2:** Accuracy, reaction times, mind-wandering ratings, and ratings of discomfort from tDCS.

		Accuracy		Reaction times		Mind-wandering ratings		Discomfort from tDCS
		Before tDCS	After tDCS	Before tDCS	After tDCS	Before tDCS	After tDCS	
F	mPFC	48.83 (0.37)	48.58 (0.34)	779.58 (28.45)	805.56 (36.10)	45.62 (6.30)	48.17 (9.26)	1.33 (0.14)
	occipital	46.42 1.99)	46.58 (2.19)	873.33 (38.29)	903.49 (57.93)	58.19 (5.40)	57.02 (5.23)	1.58 (0.19)
	sham	49.17 (0.21)	48.75 (0.30)	882.04 (66.18)	916.96 (57.17)	47.75 (8.18)	52.10 (8.29)	1.17 (0.11)
M	mPFC	49.67 (0.19)	49.50 (0.19)	850.23 (32.42)	865.02 (29.7)	55.39 (5.43)	45.43 (8.67)	1.42 (0.19)
	occipital	48.67 (0.36)	48.08 (0.68)	828.19 (34.02)	886.69 (47.24)	46.52 (9.50)	55.96 (10.67)	1.33 (0.14)
	sham	49.08 (0.23)	48.33 (0.43)	872.79 (39.21)	920.61 (56.29)	54.77 (7.23)	67.52 (6.87)	1.17 (0.11)

Note. F = females; M = males; mPFC = participant group receiving cathodal tDCS on the medial prefrontal cortex; occipital = participant group receiving cathodal tDCS on the occipital cortex; sham = participant group receiving sham stimulation. Values in parentheses are standard errors of the mean.

#### Mind-wandering ratings and content

Mean mind-wandering ratings collected during the CRT task were calculated separately for each participant before and after the stimulation (see Table 2). The Stimulation (mPFC, occipital, sham) x Gender (male, female) x Session (before tDCS, after tDCS) ANOVA on mean mind-wandering ratings showed a significant Stimulation x Session interaction (F_(2,66)_ = 3.30, p = 0.043, partial η^2^ = 0.091), qualified by a significant Stimulation x Gender x Session interaction (F_(2,66)_ = 3.49, p = 0.036, partial η^2^ = 0.096). Post hoc comparisons, performed with the Fisher test, showed that mind-wandering ratings before tDCS were comparable across groups (all p values > 0.25), indicating that before tDCS participant groups had a similar (baseline) tendency to mind-wander during the CRT task. Post hoc Fisher tests also showed that tDCS over mPFC had a different effect in men and women. Indeed, in men from the mPFC group mind-wandering ratings decreased significantly from the pre-tDCS to the post-tDCS session (55.39 vs. 45.43, p = 0.043, Cohen’s d = 0.40), whereas they tended to increase in men from the sham group (54.77 vs. 67.52, p = 0.01, Cohen’s d = 0.52) and the occipital group (46.52 vs. 55.96, p = 0.055, Cohen’s d = 0.27). In contrast, in women mind-wandering ratings did not change significantly between sessions across stimulation groups (p > 0.37 in all cases). These findings hold if we add age, education, self-reported daydreaming and working memory performance (accuracy and RTs) as covariates in the ANOVA. Thus, tDCS over mPFC reduced mind-wandering in men, but had no effect in women.

**Figure 2:**
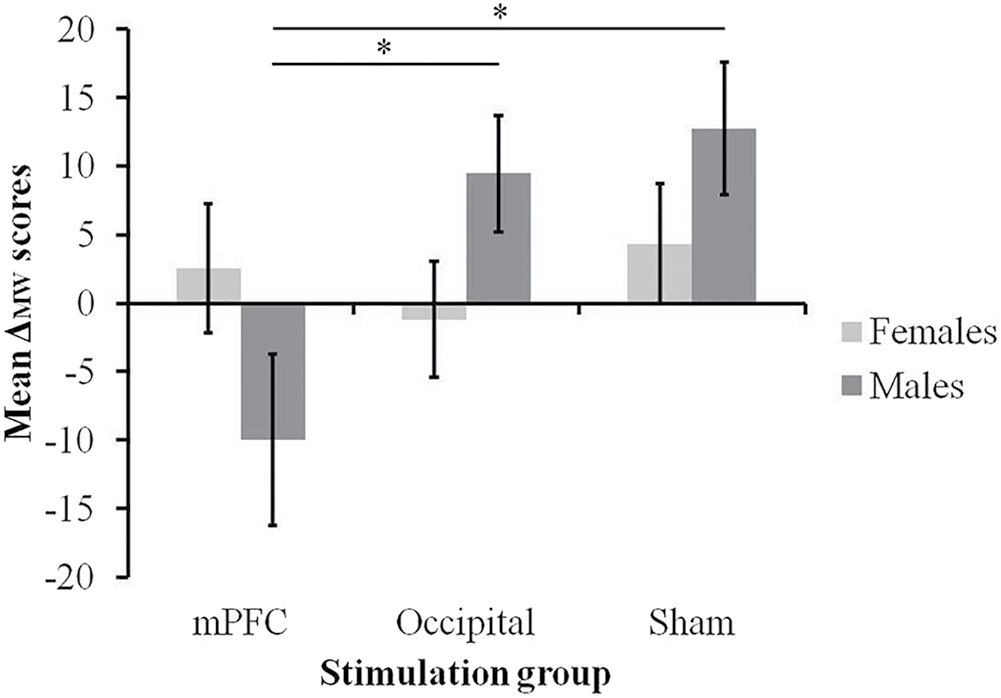
Mean *Δ-*scores for mind-wandering ratings (mind-wandering ratings post tDCS - mind-wandering ratings before tDCS) by stimulation group and gender. Error bars represent the standard errors of the mean (SEM). *p < 0.05.

To compare more directly tDCS-induced changes in mind-wandering across participant groups, we also calculated, for each participant, the difference in mean mind-wandering rating attained before and after tDCS, as *Δ*_*MW*_ = mean mind-wandering rating after tDCS – mean mind-wandering rating before tDCS (see Fig. 2). The ANOVA on *Δ*_*MW*_ with Stimulation and Gender as factors showed a significant effect of Stimulation (F_(2,66)_ = 3.30, p = 0.043, partial η^2^ = 0.091), qualified by a significant Stimulation x Gender interaction (F_(2,66)_ = 3.49, p = 0.036, partial η^2^ = 0.096). Post hoc Fisher tests confirmed a significantly reduced *Δ*_*MW*_ in men from the mPFC group compared to men in the occipital group (−9.96 vs. 9.44, p = 0.006, Cohen’s d = 1.05) and the sham group (−9.96 vs. 12.75, p = 0.001, Cohen’s d = 1.18), with no significant difference between the occipital group and the sham group (9.44 vs. 12.75, p = 0.63, Cohen’s d = 0.21). As anticipated, no effect of tDCS was observed in women, who showed comparable *Δ*_*MW*_ across the mPFC, occipital and sham groups (p > 0.42 in all cases). These findings hold if we add age, education, self-reported daydreaming and working memory performance (accuracy and RTs) as covariates in the ANOVA.

We also found sparse group differences in the content of mind-wandering and the effect of tDCS on such content (see Supplementary Table S1). In particular, in men, mind-wandering became less other-related (and relatively more self-related) with time (sham condition). This shift towards self-relatedness was significantly reduced by active tDCS (either to mPFC or the occipital cortex). No tDCS-induced differences in mind-wandering content were detected in women (Supplementary Table S1).

### Discomfort from tDCS

Discomfort ratings were non-normally distributed (Kolmogorov-Smirnov test, d = 0.43, p < 0.01), and, therefore, the data were analyzed with Kruskal-Wallis ANOVAs. We found that discomfort ratings were substantially low (M = 1.33), and similar across groups (mPFC-men, mPFC-women, occipital-men, occipital-women, sham-men, sham-women), H = 4.76, p = 0.45 (See Table 2).

## Discussion

In the current experiment we investigated the role of mPFC in mind-wandering, by comparing the propensity to mind-wander of young healthy individuals before and after receiving 2 mA cathodal monopolar tDCS over mPFC, a control site in the occipital cortex, or sham tDCS. We found that tDCS over mPFC reduced the tendency to mind-wander significantly compared to tDCS over the occipital cortex or sham tDCS, but only in men; it had no such effect in women. The reduction of mind-wandering caused by tDCS over mPFC in men was not merely an anatomically unspecific effect of administering active brain stimulation. Indeed, in participants who received tDCS over the occipital cortex we found the same tendency of mind-wandering to intensify from the pre- to the post-stimulation session observed in those who received sham tDCS, probably along with time and acquired practice with the ongoing (external) task^11,32^. tDCS over mPFC not only prevented this natural increase in mind-wandering with time, it dampened mind-wandering below baseline levels. The tDCS-induced reduction of mind-wandering aligns with fMRI evidence that mPFC is engaged during mind-wandering^10,11,21^ and neuropsychological evidence that patients with lesion to the ventral mPFC mind-wander significantly less than healthy and brain-damaged controls^23^, further pointing to mPFC as a crucial neural underpinning of mind-wandering.

One aspect of our data deserves consideration before we discuss our findings further, namely, that the predicted reduction of mind-wandering following tDCS over mPFC occurred only in men. It is unlikely that our findings were merely due to gender-related differences in general cognitive abilities known to support mind-wandering, as men and women had comparable baseline working memory capacity, performance in the CRT task, and self-reported daydreaming in daily life. A growing literature, however, shows that brain stimulation induces highly variable effects across individuals^27,33–36^, and that it can affect men and women differently^37–40^, possibly due to gender differences in brain structure^41,42^, function^43–45^, and state at the time of stimulation (i.e.,^46–49^). Compared to women, men show lower grey matter density in the orbital gyrus and the parahippocampal gyrus, and higher grey matter density in the middle frontal gyrus and posterior cingulate gyrus^50^. Moreover, men have enhanced within-hemispheric connectivity but reduced between-hemispheric connectivity compared to women^42^. Men and women also differ in the functional connectivity of resting-state networks, including the default network^43,44^. For example, men exhibit weaker connectivity than women within the default network^44,45^, especially in frontal and temporal cortices^43^. One possibility, then, is that gender differences in the structural and functional organization of distributed brain networks governing mind-wandering rendered men and women differentially susceptible to the perturbation of a specific component of these networks, as is mPFC. Future studies combining tDCS with brain imaging techniques might identify more directly differences in brain network organization that are associated with tDCS-related behavioral changes in men and women. Additionally, it is well known that the effects of brain stimulation depend on brain activity patterns in the targeted network at the time of stimulation (brain state)^46,49,51,52^. In this respect, we note that although men and women had comparable baseline ratings of mind-wandering at the CRT task, mind-wandering tended to increase over testing sessions in men (sham and active control conditions) but not in women. This finding suggests gender differences in brain activity underlying mind-wandering at the time of tDCS administration, which may have resulted in a different impact of tDCS on mind-wandering in men and women. Novel techniques to fine-tune the parameters of brain stimulation to brain state (reviewed in^49^) may prove particularly useful to test the different link between mPFC and mind-wandering observed in men and women.

The finding that cathodal tDCS over mPFC reduced mind-wandering (in men) indicates that mPFC is a crucial neural substrate of mind-wandering. As anticipated, this is not the first evidence that tDCS over prefrontal cortex can modulate mind-wandering. Axelrod and colleagues have shown increased propensity to mind-wander following anodal (supposedly excitatory) tDCS over the dorsolateral prefrontal cortex (dlPFC)^19^ (see also^20^). Axelrod *et al*.’s study and ours converge in showing that prefrontal cortex regions are crucial for mind-wandering. One important question is whether the contributions of mPFC and dlPFC during mind-wandering can be differentiated. In Axelrod *et al*.’s study, in addition to mind-wandering, tDCS over dlPFC enhanced performance in the external task, leading the authors to propose that dlPFC stimulation had improved the executive system^19^. The executive system, indeed, is thought to support some components of mind-wandering^32,53^ (see also^2^). In the present study, we found no such modulation of performance in the external task, as expected considering that mPFC does not take part in the executive network. Along with the posterior cingulate cortex, indeed, mPFC forms the ‘midline core’ of the default network^17^, a set of brain regions supporting spontaneous (as opposed to goal directed) cognition^17^. Midline core regions interact with the dorsal-medial subsystem and the medial-temporal subsystem of the default network depending, respectively, on the self-relevance and the temporal frame of constructed mental experiences^17^. Even though we are aware that the effect of tDCS is not focal, and therefore the stimulation we applied to mPFC likely reached its ventral portion, we note that tDCS-induced perturbation of mPFC had a different effect on mind-wandering than damage to ventral mPFC. Ventral mPFC damage reduced off-task thoughts related to the future^23^, consistent with the involvement of ventral mPFC in the medial temporal subsystem of the default network^17^. Cathodal tDCS of mPFC did not alter the temporality of task-unrelated thoughts, but their self-relatedness, consistent with the role of mPFC in supporting self-related processing^13,54^. We argue, therefore, that mPFC may support mind-wandering by promoting the construction of self-related, personally relevant thoughts capable to divert attention away from the current (external) task toward inner experience. A decrease in self-relatedness of task-unrelated thought, however, was observed even after tDCS of the occipital cortex. It remains to establish whether this reflects an unspecific effect of active tDCS on self-related processing, or the involvement of the occipital cortex (along with mPFC) in mediating self-related^55^ and egocentric perspectives^56^, detected in recent fMRI studies.

An alternative possibility is that tDCS over mPFC reduced meta-awareness, i.e. one’s explicit knowledge of the current contents of thought, and, in turn, the frequency with which individuals became aware of (and reported) mind-wandering^1^. Indeed, mPFC has long been implicated in meta-awareness of one’s own mental contents^57,58^. Interestingly, gender-related differences have been noted in self-monitoring^59^, which may relate to the differential effect of tDCS on mind-wandering we observed in men vs. women. Future studies using indirect (e.g., physiological) indices of mind-wandering will help clarify the degree to which lack of meta-awareness contributed to reduced mind-wandering following tDCS of mPFC.

To conclude, we have found that cathodal (supposedly inhibitory) tDCS of mPFC - but not of a control site in the occipital cortex - reduced the propensity to mind-wander. This effect, however, was found only in men. These findings indicate that mPFC is crucial for mind-wandering, possibly by enabling construction of self-relevant scenarios capable to divert attention away from perceptual reality towards inner contents. Gender-related differences in tDCS-induced changes, however, suggest that mPFC controls mind-wandering differently in men and woman, possibly due to differences in the structural and functional organization of distributed brain networks including mPFC, which orchestrate collectively the natural alternation of the human mind between states of focused attention and mind-wandering.

## Methods

### Participants

Seventy-two healthy right-handed participants with no history of neurological or psychiatric disease participated in this study (for demographic information, see Table 1). Participants were randomly assigned to three stimulation groups: the mPFC group (N = 24; to receive tDCS over mPFC; see below), the occipital group (N = 24; to receive tDCS over occipital cortex), and the sham group (N = 24; to receive sham tDCS). Each stimulation group was composed by 12 women and 12 men so to form a total of 3 (Stimulation) × 2 (Gender) = 6 groups of participants. Participants were blind to the type of stimulation they were going to receive. Two Stimulation (mPFC, occipital, sham) x Gender (female, male) analyses of variance (ANOVA) on age (all F < 2.81, all p > 0.06) and years of education (F < 0.53, p > 0.59 in all cases) revealed no significant effects or interactions, though age was slightly higher in males relative to females (23.3 vs. 23.0 years) across stimulation groups (Table 1). Participants gave written informed consent to participate to the study, which was approved by the Bioethical Committee of the University of Bologna and carried out in agreement with legal requirement and international norms (Declaration of Helsinki 1964). The methods carried out in this work are in accordance with the approved guidelines.

### Procedure

#### Preliminary meta-analysis of neuroimaging studies

To select the optimal brain coordinates to target mPFC, we conducted a preliminary activation likelihood estimation (ALE) meta-analysis (see^25,26^) of fMRI studies of mind-wandering aimed at individuating the mPFC subregion most reliably associated with mind-wandering. We conducted a search of the literature using PUBMED (www.ncbi.nlm.nih.gov/pubmed) for papers containing the words “mind-wandering”; “spontaneous thought”; “stimulus-independent thought”; “task-unrelated thought”; or “daydreaming” (concluded on May 31, 2014). Included papers had to: (1) report functional neuroimaging studies (i.e., fMRI or PET), (2) report specific peak foci of activation in a standard reference frame (MNI or Talairach space), (3) report group results, (4) involve healthy participants, and (5) employ an explicit measure of spontaneous thoughts (e.g., online reports, retrospective reports, questionnaires). We considered contrasts of conditions conducive to mind-wandering (e.g., rest) vs. on-task thought, or correlation analyses between degree of mind-wandering and brain activity.

We excluded studies that did not distinguish between mind-wandering and task-related thought (e.g.^60^). We also excluded the study by D’Argembeau *et al*., in which rest was compared to (directed) internal thinking^61^. Given that spontaneous and directed forms of internal cognition involve highly similar processes and brain regions, brain regions supporting mind-wandering were probably subtracted out in the reported contrast (see also^22^). For papers showing more than one experiment with independent samples, each experiment was considered individually (e.g.^62^). Table 3 recapitulates the studies included.

**Table 3:** Studies included in the ALE meta-analysis.

Study	N	Mind wandering report	Contrast/Analysis
a. Mcguire *et al*. 1996^62^ – Study 1	5	Retrospective	Correlation between stimulus-independent thought frequency and regional cerebral blood flow
b. Mcguire *et al*. 1996^62^ – Study 2	6	Retrospective	Correlation between stimulus-independent thought frequency and regional cerebral blood flow
c. Binder *et al*. 1999^67^	30	Inferential	Rest vs. Tone task
d. Mason *et al*. 2007^11^	19	Inferential and questionnaire	'Practiced > novel’ inclusively masked with ‘baseline > all tasks’; Correlation between frequency of mind-wandering and the change in BOLD signal observed when participants performed ‘practiced’ relative to ‘novel’ blocks
e. Christoff *et al*. 2009^10^	15	Online	Intervals prior to off-task reports vs. intervals prior to on-task reports
f. Wang *et al*. 2009^68^	12	Questionnaire	Correlation between regional homogeneity reflected activity and spontaneous thought processes frequency during resting state
g. Dumontheil *et al*. 2010^58^	16	Retrospective	Low demanding tasks vs. High demanding tasks
h. Stawarczyk *et al*. 2011^12^	22	Online	Mind wandering > on-task; External distraction > on-task; Task related interferences > on-task
i. Hasenkamp *et al*. 2012^69^	14	Online	Awareness of mind wandering > mind wandering; Mind wandering > shift
j. Kucyi *et al*. 2013^70^	51	Online	Attention to something other than pain > Attention to pain
k. McKiernan *et al*. 2006^71^	30	Inferential	Correlation between task-induced deactivation and task-unrelated thought frequency
l. Fransson, 2006^72^	14	Retrospective	Rest > Working memory task

Note. N = sample size; Mind wandering report = how mind wandering experiences were collected; condition = experimental condition added to the ALE meta-analysis.

A quantitative, random-effects meta-analytic method known as activation likelihood estimation (ALE) was conducted (see^25,26^), using the software GingerALE, Version 2.3 (http://brainmap.org/ale/). The ALE algorithm tests for above-chance clustering of peak foci from different experiments included in the meta-analysis by comparing actual activation foci locations/clustering with a null distribution that includes the same number of peak foci distributed randomly throughout the brain’s gray matter. Resulting statistical maps show clusters where convergence between activation foci is greater than would be expected by chance. A non-additive ALE method was chosen to restrict the number of inflated ALE values resulting from contrasts with many closely located activation foci^26^. An advantage of this method is to reduce the risk of within-experiment effects rather than the between-experiment concordance to be the cause of significant ALE values^26^. Statistical maps were thresholded using a false discovery rate (FDR) of q = 0.01 and minimum volume of 50 mm^3^. We found that the largest cluster in prefrontal cortex was located in BA 10 (mm^3^ = 1368), with center of mass in x = −6.15, y = 49.31, z = −2.4 (Talairach space), followed by smaller clusters in BA 9 (mm^3^ = 152), BA 32 (mm^3^ = 88), BA 10 (mm^3^ = 88), and BA 9 (mm^3^ = 56). The coordinates of the largest BA 10 cluster were used to derive the position of the mPFC scalp electrode in the tDCS experiment (see below).

#### tDCS

tDCS was delivered using a battery-driven Eldith constant direct current stimulator (neuroConn GmbH). A pair of surface sponge electrodes was soaked in a standard saline solution (NaCl 0.9%) and held in place with elastic rubber bands. In all participants, a monopolar tDCS montage was used, with the cathodal (5 × 5 cm) and anodal (5 × 7 cm) electrodes placed over a scalp region and the right deltoid, respectively. Participants were randomly assigned to receive either active cathodal stimulation over the mPFC (mPFC group), active cathodal stimulation over the occipital cortex (occipital group, serving as the active stimulation control group), or sham stimulation (sham group). Active tDCS was delivered with a constant current of 2 mA (current density ~0.08 mA/cm^2^), complying with current safety guidelines^28^. Stimulation lasted for 15 min, plus 15 s of ramp-up and ramp-down at the beginning and end of stimulation. Impedance was constantly monitored and kept below 8 kOhm. This protocol is known to affect cortical excitability for more than 30 min after the end of stimulation^63^, thus covering the entire duration of the testing phase.

In the mPFC group, the location of the cathode was chosen based on the results of the ALE meta-analysis, showing consistent activation of BA10 during mind-wandering, with center of mass in x = −6.15, y = 49.31, z = −2.4 (Talairach space). These coordinates were transformed into 10–20 electroencephalography system coordinates using the Münster T2T-converter software (www.neuro03.uni-muenster.de/ger/t2tconv/), and the cathode was placed on the corresponding position on the scalp, slightly to the left of Fpz (Fig. 1). The use of an extracephalic montage ruled out potential confounding effects of a cephalic reference^27,29^. With this montage, the current travels ventrally from the frontal surface to the right arm, thereby most probably affecting also the ventral portion of mPFC^37^. In the occipital group, the cathode was placed over the occipital cortex (10–20 system: Oz) and the anode was placed over the right deltoid muscle, as in the mPFC group. In the sham group, the electrodes were placed in the same positions as in the mPFC group, but the stimulator was turned off after 30 seconds of cathodal stimulation. Thus, participants felt the initial itching sensation associated with active tDCS, but they received no current for the rest of the “stimulation” period. This procedure ensures successful blinding of participants^64^. Although the intensity used in our study (2 mA) may be less effective in ensuring blinding^65^ (but see^27^), we used relatively small cephalic electrodes to reduce scalp sensations, and make active and sham stimulation feel comparable^66^. Immediately after the stimulation, participants in all groups rated on a 5-point Likert scale the discomfort experienced during the stimulation (from 1 – ‘no discomfort’ to 5 – ‘extreme discomfort’).

### Tasks

#### Daydreaming

Before starting the experimental session, participants completed the daydreaming frequency subscale of the Imaginal Processes Inventory (IPI)^30^, a questionnaire designed to examine individual differences in inner mental life. In a series of 12 daydreaming items, individuals rated the extent to which they experienced mind-wandering in their daily life (e.g., “Whenever I have time on my hand I daydream”, from 1 – ‘never’ to 5 – ‘always’). The score ranges from 12 to 60, with higher scores indicating a higher propensity toward daydreaming.

#### Working-memory

After completing the daydreaming frequency subscale, all participants were evaluated on working memory, an aspect of executive functioning that may have an impact on mind-wandering^32,53^. Working memory was assessed with a 3-back task^31^, which requires to monitor a series of 100 digit stimuli (from 1 to 8), and signal whether the number currently presented matches the number presented three trials back. Each number was presented for 2 s, followed by a fixation cross lasting 1.5 s. Correct responses, false alarms, and reaction times were recorded.

#### Experimental assessment of mind-wandering

Participants then underwent a choice reaction time (CRT) task modified from previous studies^14,23^. In the CRT task, individuals saw a stream of digits (1–8) appearing in the center of the screen, written in black ink (non-targets) or in green ink (targets). A total of 312 non-targets and 50 targets were presented (see also^14^). Upon presentation of a green digit, participants had to report whether that number was even or odd using one of two response buttons. Non-target and target stimuli were arranged in ten blocks containing approximately 5 targets and 31 non-targets each, whose order was randomized for each participant (see also^23^). Presentation rate was 1 item every 1.5 s for non-targets and 1 item every 2 s for targets, followed by a 2 s fixation cross. Mind-wandering was assessed through the presentation of 10 thought probes, one for each block. Thought probes were presented visually, as a series of three screens. First, participants were required to rate, on a Visual Analog Scale (VAS), the degree to which, immediately before the probe, their attention was on-task, i.e., focused on the task being performed, vs. off-task, i.e., focused on something unrelated to the task (from 0 – ‘completely on-task’, 100 – ‘completely off-task’). If participants had, to some extent, experienced task-unrelated thoughts (trials receiving a VAS rating > 0), they were required to classify their thoughts into one of 6 categories: 1) Past (i.e., the thought pertained to the past; e.g. “The holiday in Rome was the best”), 2) Present (i.e., the thought pertained to the present; e.g. “I wonder what my girlfriend is doing now”), 3) Future (i.e., the thought pertained to the future; e.g. “I am seeing Alessandro later”), 4) External distractions (i.e., the thought pertained to distractions from the external environment, e.g., “Was that a thunder?”), 5) Time not clear (i.e., the thought was not easily classified into time categories, e.g. “I’m lucky to have a friend like him”), or 6) Unaware (i.e., the participant is not aware of the content of her/his thought). In a following screen, participants additionally specified whether their task-unrelated thoughts were 1) Self-related (i.e., the thought mainly pertained to the self; e.g. “I am so going to the gym after this”), 2) Other-related (i.e., the thought mainly pertained to other people; e.g. “My dad is getting old”), or 3) Unrelated to people (i.e., the thought did not involve people; e.g. “The new car was a good deal”). The CRT task and concomitant assessment of mind-wandering were performed both before and after the tDCS or sham stimulation session.

### Data availability

The datasets generated during and/or analysed during the current study are available from the corresponding author on reasonable request.

## Electronic supplementary material

Supplementary Table S1
