# Supplementary material for: Transcranial direct current stimulation of the medial prefrontal cortex dampens mind-wandering in men
Source: Sci Rep. 2017 Dec 5;7:16962. doi: 10.1038/s41598-017-17267-4 (PMC5717259; doi:10.1038/s41598-017-17267-4)
Supplement: Supplementary file 1 — Supplementary Table S1 [file 41598_2017_17267_MOESM1_ESM.doc]

SUPPLEMENTARY INFORMATION

Transcranial direct current stimulation of the medial prefrontal cortex dampens mind-wandering in men

Elena Bertossi^1^, Ludovica Peccenini^1^, Andrea Solmi^1^, Alessio Avenanti^1,2^, and Elisa Ciaramelli^1,*^

^1^ Dipartimento di Psicologia, Università di Bologna, Bologna, Italy

^2^ IRCCS Fondazione Santa Lucia, Roma, Italy

*Corresponding author:

Elisa Ciaramelli, PhD

Dipartimento di Psicologia, Università di Bologna

Viale C. Berti-Pichat 5, 40126 Bologna, Italy

Phone number: +39 0547 338951

Fax number: +39 0547 338952

Email: elisa.ciaramelli@unibo.it

**Supplementary Table S1**. Percentage of different contents of mind-wandering before and after tDCS

|  |  | Past | | | Present | |  | Future | | | Distractions | | | Time not-clear | | | Unaware | | | Self | | | Other | | | None | | |
| --- | --- | --- | --- | --- | --- | --- | --- | --- | --- | --- | --- | --- | --- | --- | --- | --- | --- | --- | --- | --- | --- | --- | --- | --- | --- | --- | --- | --- |
|  |  | Before | After | *Δ* | Before | After | *Δ* | Before | After | *Δ* | Before | After | *Δ* | Before | After | *Δ* | Before | After | *Δ* | Before | After | *Δ* | Before | After | *Δ* | Before | After | *Δ* |
| F | mPFC | 8.6  (2.5) | 11.0  (4.2) | 2.4  (4.5) | 19.7  (4.0) | 22.1  (4.5) | 2.4  (4.7) | 25.6  (4.7) | 22.7  (5.3) | -3.0  (5.0) | 26.5  (6.1) | 18.1  (6.7) | -8.3  (4.1) | 16.2  (6.0) | 21.9  (4.8) | 5.7  (4.0) | 3.3  (1.9) | 4.2  (1.9) | 0.8  (1.9) | 43.7  (6.4) | 44.3  (5.3) | 0.6  (7.6) | 38.5  (4.1) | 42.0  (5.4) | 3.5  (8.1) | 14.4  (4.4) | 9.5  (4.0) | -4.9  (3.5) |
|  | occipital | 13.3  (4.3) | 7.5  (3.5) | -5.8  (3.4) | 28.3  (4.9) | 23.3  (4.8) | -5.0  (6.9) | 15.8  (3.1) | 26.7  (5.3) | 10.8  (5.1) | 18.3  (3.7) | 13.3  (3.6) | -5.0  (4.8) | 16.7  (4.5) | 17.5  (3.0) | 0.8  (4.0) | 7.5  (2.2) | 11.7  (4.4) | 4.2  (4.0) | 35.8  (4.8) | 40.8  (8.0) | 5.0  (8.1) | 25.8  (4.2) | 24.2  (3.6) | -1.7  (3.4) | 30.8  (6.0) | 23.3  (4.7) | -7.5  (5.4) |
|  | sham | 16.8  (3.4) | 17.2  (3.7) | 0.4  (5.7) | 19.1  (6.0) | 14.5  (3.4) | -4.6  (6.3) | 28.6  (4.9) | 33.2  (5.1) | 4.5  (5.2) | 15.9  (4.3) | 11.8  (3.4) | -4.2  (4.3) | 17.9  (4.6) | 17.5  (3.6) | -0.4  (4.5) | 1.7  (1.1) | 5.9  (2.6) | 4.3  (2.0) | 35.6  (5.5) | 32.7  (4.2) | -2.9  (4.5) | 49.8  (4.8) | 44.2  (6.1) | -5.6  (6.4) | 12.9  (3.2) | 17.2  (4.4) | 4.3  (5.7) |
| M | mPFC | 14.4  (4.7) | 10.9  (4.0) | -3.4  (3.6) | 30.4  (5.4) | 22.9  (6.3) | -7.5  (7.1) | 24.5  (4.9) | 21.0  (6.6) | -3.4  (4.2) | 12.7  (2.8) | 9.4  (2.7) | -3.3  (3.2) | 12.9  (3.1) | 25.3  (5.0) | 12.4  (4.6) | 5.2  (2.4) | 10.6  (5.6) | 5.4  (6.0) | 43.6  (3.7) | 43.3  (8.7) | -0.3  (7.4) | 31.2  (3.6) | 24.5  (4.7) | -6.7  (4.1) | 20.0  (4.4) | 21.6  (6.1) | 1.6  (5.3) |
|  | occipital | 10.7  (4.5) | 8.1  (3.1) | -2.6  (5.7) | 31.7  (6.0) | 12.8  (4.2) | -19.0  (7.9) | 14.6  (5.1) | 23.1  (7.0) | 8.4  (7.9) | 27.4  (4.8) | 28.9  (10.5) | 1.5  (8.5) | 12.2  (4.0) | 25.6  (7.9) | 13.3  (8.2) | 3.3  (1.4) | 1.7  (1.7) | -1.7  (1.7) | 34.7  (4.3) | 25.4  (8.2) | -9.3  (7.2) | 37.1  (5.7) | 40.0  (9.8) | 2.9  (8.6) | 24.9  (6.2) | 32.9  (8.1) | 8.0  (5.2) |
|  | sham | 11.8  (5.5) | 14.3  (4.0) | 2.5  (5.8) | 16.2  (3.6) | 10.9  (2.9) | -5.3  (3.8) | 22.9  (4.3) | 25.4  (5.0) | 2.5  (6.1) | 23.4  (6.2) | 19.3  (4.3) | -4.1  (3.5) | 20.6  (5.6) | 21.9  (4.6) | 1.2  (4.4) | 5.1  (1.5) | 8.3  (3.0) | 3.2  (2.3) | 25.6  (5.5) | 34.4  (5.8) | 8.8  (5.1) | 42.8  (6.1) | 18.7  (3.9) | -24.1  (6.2) | 26.4  (7.2) | 38.5  (7.4) | 12.1  (8.0) |

Note. F = females; M = males; mPFC = cathodal stimulation of medial prefrontal cortex; Occipital = cathodal stimulation of occipital cortex; *Δ* = difference between pre- and post-tDCS. In parenthesis we report the standard errors of the mean. To investigate whether tDCS over mPFC modulated the content of mind-wandering, we first counted the number of times participants described the contents of their thoughts as belonging to different categories (past, present, future, current distractions, time not clear, unaware, self-related, other-related, and unrelated to people). We then computed the ratio between the number of thoughts for each content category and the total number of mind-wandering episodes claimed (trials receiving a VAS rating > 0), thus obtaining an index of the ‘quality’ of mind-wandering independent of quantity, separately for the pre- and post-tDCS sessions, which we report, for space reasons, as percentages. We first verified whether there were group differences in the contents of mind-wandering before tDCS. A Kruskal-Wallis ANOVA on the frequency of other-related thoughts with Group as factor (mPFC-men, mPFC-women, occipital-men, occipital-women, sham-men, sham-women) revealed an effect of group (H = 12.01, p = 0.03). There were no significant group differences in the other content categories (H < 8.73, p > 0.12 in all cases). We followed-up the effect of group running separate Kruskal-Wallis ANOVAs in men and women. In women, the ANOVA detected a significant effect of stimulation (H = 8.54, p = 0.01), such that the sham group experienced a higher proportion of other-related thoughts than the occipital group before tDCS (0.498 vs. 0.258, z = 2.77, p = 0.005). In men, the same ANOVA detected no significant difference among stimulation groups (H = 2.71, p = 0.26). Next, we calculated *Δ*-scores as content ratio after the stimulation – content ratio before the stimulation, for each content category and each participant. A Kruskal-Wallis ANOVA on *Δ*-scores for other-related thoughts with Group as factor (mPFC-men, mPFC-women, occipital-men, occipital-women, sham-men, sham-women) revealed an effect of group (H = 12.30, p = 0.03), while group differences in *Δ*-scores for the other content categories were not significant (H < 7.22, p > 0.20 in all cases). A Kruskal-Wallis ANOVAs on *Δ*-scores for other-related thoughts in men showed a significant effect of stimulation group (H = 7.86, p = 0.02), with a higher *Δ_OTHER_* in the mPFC group (-0.067 vs. -0.241, z = 2.11, p = 0.03) and in the occipital group compared to the sham group (0.029 vs. -0.241, z = 2.42, p = 0.01), but no difference between the mPFC and the occipital group (p = 0.22). We note that in the sham group the decrease in other-related thoughts in the post- (compared to the pre-) tDCS session (before: 0.428 vs. after: 0.187, Wilcoxon test z = 2.85, p = 0.004) came along with a marginal increase in self-related thoughts (before: 0.256 vs. after: 0.344, Wilcoxon test: z = 1.75, p = 0.08), not observed in the mPFC group and in the occipital groups (p > 0.27 in both cases). The same ANOVA in women yielded no significant difference among stimulation groups (H = 1.48, p = 0.48). Thus, in men, mind-wandering became less other-related (and relatively more self-related) with time (sham condition), and this shift towards self-relatedness was significantly reduced by active tDCS (either to mPFC or the occipital cortex).
